# Investigating the knowledge and preparedness of proximal residents to a general-emergency event at the Koeberg Nuclear Power Station

**DOI:** 10.4102/jamba.v8i2.208

**Published:** 2016-01-13

**Authors:** Alberto P. Francioli

**Affiliations:** 1 Department of Geography and Environmental Studies, Stellenbosch University, South Africa

## Abstract

Ward 23 in the City of Cape Town South Africa is situated within 16 km of Koeberg Nuclear Power Station (KNPS). Massive investments were made to provide the 13 800 residents of Ward 23 with information on emergency preparedness and evacuation procedures in case of a general-emergency event at KNPS. However, it is not known whether these efforts to inform and prepare the proximal residents for a general emergency have been effective or not. The objective of this study was to investigate the level of knowledge of and preparedness for an emergency exhibited by residents of Ward 23. Data was collected through the distribution of semi-structured questionnaires to 204 residents at the study site to ascertain their views on the provision and accessibility of emergency information and to find out whether they utilised this information to prepare themselves. The results revealed that the majority of interviewed residents had very poor knowledge concerning emergency procedures, and few had made any effort to prepare themselves. The majority of better-informed and prepared residents tended to reside closer to KNPS. The poor levels of knowledge and preparedness are attributed to residents’ lack of awareness concerning the availability of information, misconceptions regarding a nuclear hazard and a general emergency event or a lack of incentive to inform themselves due to a perceived high sense of security and the improbability of such an event occurring. To improve resident knowledge and preparedness, efforts should be made to advertise the availability and importance of such emergency information and enhance incentives for residents to inform themselves.

## Introduction

Nuclear energy, whilst being a critical source of energy, also has the potential to be a devastating hazard (Koronowski & Romm 2013). Over the last 40 years, there have been several general emergency events, causing significant disturbances to people’s health and livelihoods (World Nuclear Association 2012). During such events, it has been necessary to evacuate people living within the vicinity of such stations in order to avoid contamination by radiological fallout. However, residents and disaster-management institutions have shown during many of these past evacuations that they are not well aware of and prepared for such hazards (Funabashi & Kitazawa 2013; Ziegler & Johnson 1984). These hazards caused massive confusion and uncertainty amongst evacuees, which created greater disorder, obstructed mitigation efforts and consequently put thousands at risk of exposure to a radiological hazard.

The Koeberg Nuclear Power Station (KNPS) is situated approximately 30 km from central Cape Town. Approximately 140 000 people reside within the immediate 16 km radius of the KNPS, known as the Formal Emergency Planning Zone (FEPZ). Institutions such as Eskom and the City of Cape Town Disaster Risk Management Centre (CoCT DRMC) invested much effort and resources into preparing for a potential general emergency. This includes developing evacuation models, integrated disaster-management plans amongst local-government institutions and emergency services as well as safety-and-preparedness guidelines for the public to inform and prepare themselves in order to reduce the risk of them being exposed to radiological hazards. However, despite the availability of this information through various mediums, it is unknown whether residents of the FEPZ utilise the information to better inform and prepare themselves for a general emergency event.

This article aims to present the findings of an exploratory research project amongst residents residing in the proximity of KNPS, investigating the levels of knowledge and preparedness concerning a general hazard. Specifically the main objectives of the article were to:

- ascertain the level of the residents’ knowledge of and familiarity with general emergency-related information, plans and procedures
- identify their attitude and perception of access to and the availability of general emergency-related information as well as whether it is beneficial to them
- examine what practices, strategies and plans residents would follow in a general emergency event
- determine whether a geographic distance-decay style of relationship exists between respondents’ levels of informedness and preparation and their geographical proximity to KNPS.

The findings of this investigation will be discussed and compared to local and international literature to interpret the results. Finally, several recommendations for stakeholders will be put forward to address the issues raised in this article.

## The literature on general emergencies and disaster preparedness

Nuclear energy has been regarded as popular source of energy generation for the last 60 years with 434 nuclear power stations operating globally at present (International Atomic Energy Agency 2013). Despite the danger associated with the radiological elements utilised to generate electricity at nuclear power stations, the high-quality engineering, workforce, management and safety guidelines involved in the operation of these facilities have allowed the nuclear-energy industry to flourish and to be regarded as a safe and reliable form of energy generation (Funabashi & Kitazawa 2013; Koronowski & Romm 2013). However, several general emergencies (in which large quantities of radiation have been released or leaked from the nuclear plant into the surrounding environment) have occurred at nuclear power stations such as Three Mile Island, United States of America (USA), in 1979 (World Nuclear Association 2012); Chernobyl, Union of Soviet Socialist Republics, in 1986 (World Nuclear Association 2013a) and recently Fukushima Dai-ichi (FNPS), Japan, in 2012 (World Nuclear Association 2013b). During these events, communities within the vicinity of these plants were under threat of exposure to and contamination by the radiological release.

Before the Three Mile Island or Chernobyl disasters, the improbability of a general emergency event was so high that it was considered unnecessary by most nuclear power stations or their regulatory bodies to develop any emergency or evacuation plans (Carter & Thompson 1987). Consequently, when these events occurred, evacuation and relief efforts were severely hampered due to the poor emergency management. These obstacles were exacerbated further by the massive confusion and panic amongst proximal residents who were poorly prepared to react to such hazards.

Subsequently, nuclear-related stakeholders globally put a much greater effort into formulating strategies and procedures to respond to a general emergency event (Council for Excellence in Government 2006). These include providing civilians residing in the vicinity of nuclear stations with details on emergency procedures and evacuation in order to reduce their risk and vulnerability (San Luis Obispo County 2013). This information is usually made available through mail, flyers, safety guidelines, public information centres, regular public-safety forums or online information (Nuclear Information and Resource Centre 2011; Oak Ridge Associated Universities 2010). Such strategies coincide with the recent shift in the disaster-management paradigm which increasingly emphasises strategies of disaster-risk reduction (DRR) to focus more on preventative or mitigating approaches in order to reduce the vulnerabilities of particular groups (Van Niekerk & Vermaak 2004; Van Riet 2009). According to Pelling’s (2003) theory of urban vulnerability, awareness of and preparedness for particular hazards are crucial aspects in reducing people’s level of vulnerability to any hazard.

Unfortunately, despite the previous paradigm shifts in thinking about general emergencies and disaster reduction, Japan was caught unprepared during the 2011 FNPS meltdown (Funabashi & Kitazawa 2013). For the past several decades, nuclear energy in Japan (like in many other countries) has been considered to be one of the safest power-generating means available with a very good safety track record (Tanaka 2012). However, not wanting to generate concern amongst the population or create a lack of confidence in the nuclear industry in Japan, officials did not promote disaster planning or drill practices as they believed it would cause unnecessary anxiety amongst people living near nuclear facilities (Funabashi & Kitazawa 2013). A false sense of security was largely to blame for the chaotic and disorganised manner in which people were evacuated during the 2011 FNPS general emergency. Consequently, when the disaster struck, local government and evacuation coordinators were ill prepared on how to proceed, and local residents were poorly informed and confused as to what they should do.

Along with South Africa’s national energy provider and nuclear regulator, Eskom, the CoCT DRMC has shown commitment to formulating general emergency strategies and procedures. These include several integrated plans and models to assist in preparing evacuation and disaster-response strategies in case of a general emergency (Eskom 2008; Marks *et al*. 2007). They have also shown commitment to communicating general emergency-related information to the public through various mediums such as Information Calendars (Eskom 2013c), Family Disaster Preparedness Guidelines (Disaster Risk Management – Cape Town 2012), a quarterly Public Safety Information Forum (Eskom 2012) as well as information online and in public libraries (City of Cape Town 2012a; Eskom 2013a, 2013b).

However, the recent general emergency in Japan has brought about a rise in concern about and criticism against the KNPS (Froggatt, Hazemann & Schneider 2012; Koeberg Alert Alliance 2012; Phakathi 2012). Immediately after the incident, media and civil-society groups raised questions about KNPS’s safety and preparedness for a general emergency, especially in light of the realisation that the station was built within 8 km of a fault line (Gosling 2011; Raubenheimer 2013). Concern has also been raised regarding the rapid growth of suburbs and development within the FEPZ and whether evacuation plans have taken into consideration the increasing size of the population (Disaster Risk Management – Cape Town 2012; Marks *et al*. 2007).

## Methodology

### Site and respondent selection

Ward 23 forms part of the City of Cape Town District Municipality, lying approximately 12 km north of central Cape Town and directly south of the KNPS. According to South African census data from 2011, the ward’s population is approximately 34 000 (Department of Strategic Development Information and GIS 2013). Other key demographic information is as follows:

- The predominant race is white (European) South African (76%).
- Of those aged 20 years and older, 82% have completed high school and some form of tertiary education (45%).
- Of the labour force (ages 15 to 64), 95% is employed.
- Concerning income, 83% of the population earn above ZAR 3200 per month. Furthermore, 21.5% of the ward’s population earn between ZAR 12 801 and 25 600, and 22.8% earn between ZAR 25 601 and 51 200.

**FIGURE 1:**
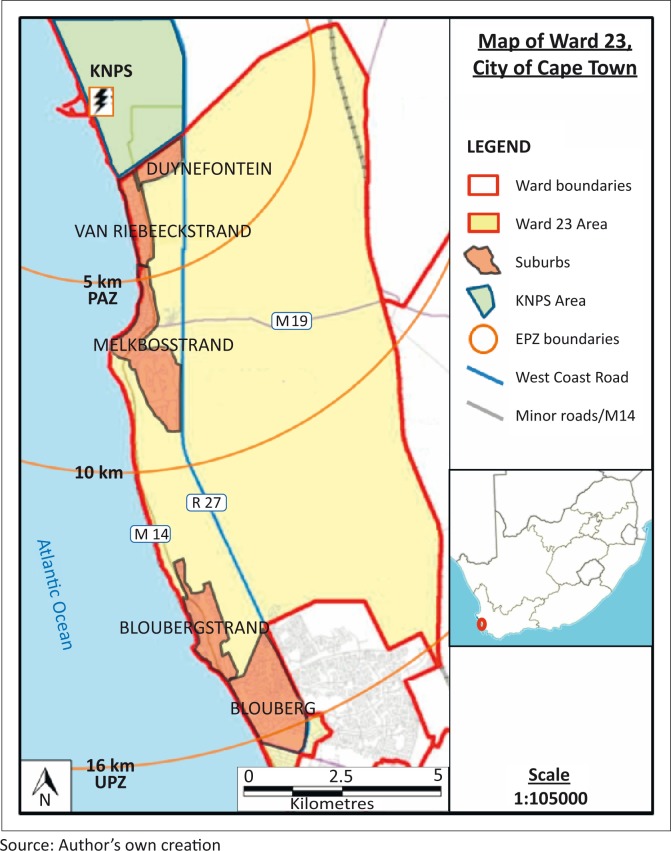
Map of study site, Ward 23.

The majority of the ward’s area is farmland or undeveloped land (City of Cape Town 2012b) with most of the population and development concentrated along the coast in five distinct suburbs, namely Blouberg, Bloubergstrand, Melkbosstrand, Van Riebeeckstrand and Duynefontein (see Figure 1).

Whilst development has been kept to a minimum due to the proximity of KNPS, development along the coast has grown rapidly. Between 2001 and 2011, the number of households and the population grew by 75% and 70%, respectively. Ward 23 was selected as the study site because the ward is situated almost wholly within KNPS’s 16 km Formal Emergency Planning Zone with its five suburbs situated next to the station as seen in Figure 1 and Table 1.

**TABLE 1:** Approximate distances between Koeberg Nuclear Power Station and the suburbs in Ward 23.

Suburb	Approximate suburb distance from KNPS	
	Kilometres	Miles
Duynefontein	2-2.6	1.24-1.62
Van Riebeeckstrand	2.5-4.12	1.55-2.56
Melkbosstrand	4.52-6.86	2.81-5.18
Bloubergstrand	12.66-15.30	7.87-9.51
Blouberg	15.31-16.54	9.51-10.28
FEPZ	16†	9.9

FEPZ, Formal Emergency Planning Zone; KNPS, Koeberg Nuclear Power Station.

† The FNPS boundary is not a perfect 16 km radius from the KNPS, appearing ‘jagged’ to take streets and properties into account.

A purposive-sampling technique was employed by the researcher in the study site. The sampling criteria included only adults (18 years and over) who resided (or considered themselves residents) in one of the five suburbs of Ward 23. Minors (below 18 years), daily commuters or day visitors into the ward were excluded from the sampling. The respondents were divided into five geographical strata based on the five suburbs located in the study site, which would allow for:

- maximising respondent variation within the purposive sample (Koerber & McMichael 2008)
- observing any relationship or differences between respondents’ proximity to KNPS and their levels of knowledge and preparedness.

### Questionnaire layout and distribution

A semi-structured, qualitative questionnaire was developed and distributed to residents over the course of four weeks. The questionnaire consisted of 33 questions, which included open-ended, polar and Likert-scale based questions. Polar and Likert-scale type questions had an adjoining section for respondents to explain their selected answers more descriptively. The questionnaires were styled on the KAP method (World Health Organization 2008) with questions grouped into particular themes, focussing on:

- ‘Knowledge’-oriented questions assessed respondents’ awareness of and familiarity with information provided in general emergency guidelines and plans. These questions assessed how much respondents knew and the accuracy of their knowledge of the information available.
- ‘Attitude’-oriented questions focussed on analysing respondents’ perceptions and opinions on particular issues and matters.
- The ‘Practices’-oriented questions explored the measures that respondents would take in preparing themselves and their households as well as the course of action they would take in a general emergency.
- Sampling took place in each of the suburbs, the researcher going from door to door to interview residents one at a time.

### Methodology for data consolidation

A total of 230 questionnaires were completed via door-to-door distribution. Questionnaires with errors, incomplete answers, inconsistencies, lack of depth or ‘prank answers’ were removed from the data consolidation. In total, 203 questionnaires were acceptable for consolidation (Table 2).

**TABLE 2:** Total number of questionnaires consolidated for findings.

Ward 23 suburbs	Total number of questionnaires consolidated
Duynefontein	33
Van Riebeeckstrand	37
Melkbosstrand	50
Bloubergstrand	31
Blouberg	52

Data have been consolidated and are presented in a qualitative and descriptive format in the ‘Analysis of findings’ section. Some data were quantified into charts in order better to examine and display response proportions and variation between different suburbs. A discussion of the consolidated data will be presented in the following section. The data was compared with relevant literature.

## Analysis of findings

### Analysis of respondents knowledge

#### Awareness of information

**FIGURE 2:**
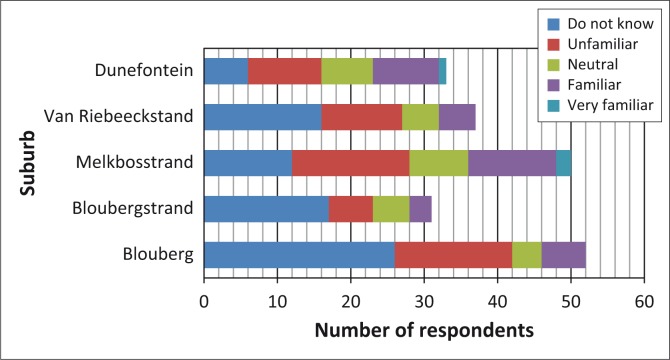
Respondents’ familiarity with information related to general emergencies.

Almost half of respondents in Ward 23 were unaware of general emergency-related information or quarterly forums that have been made available to the public (Figure 2). This was observed especially in the communities of Blouberg and Bloubergstrand where the majority of respondents claimed to be unaware of any sources of information (47 of 52 respondents and 25 of 31 respondents, respectively). A greater proportion of the respondents from Melkbosstrand and Duynefontein appeared to be aware of the existence of information related to general emergencies.

#### Knowledge of and familiarity with information and protocol about general emergencies

**FIGURE 3:**
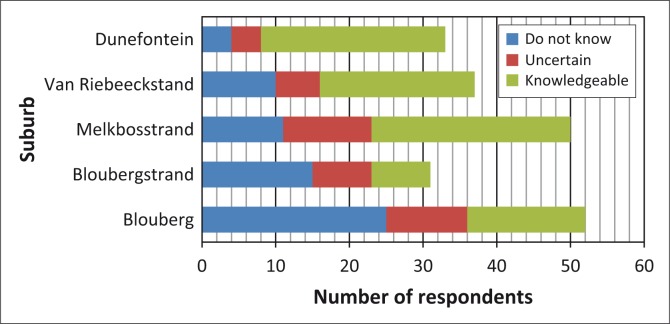
Respondents’ knowledge of methods of general-emergency notification.

**FIGURE 4:**
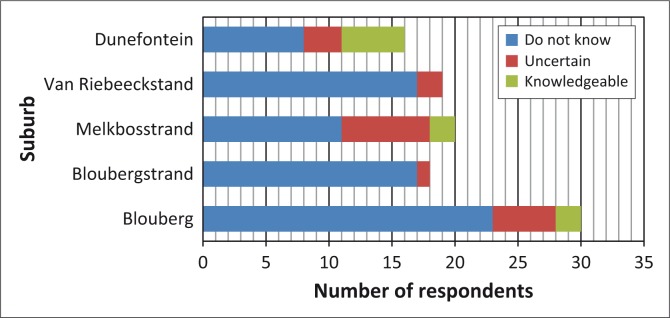
Respondents’ knowledge of procedure for evacuating school children.

**FIGURE 5:**
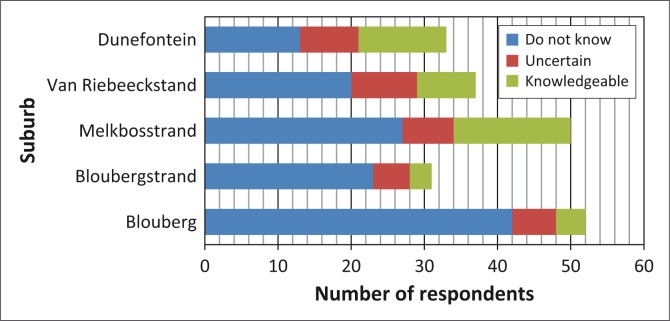
Respondents’ knowledge of evacuation routes out of Formal Emergency Planning Zone.

**FIGURE 6:**
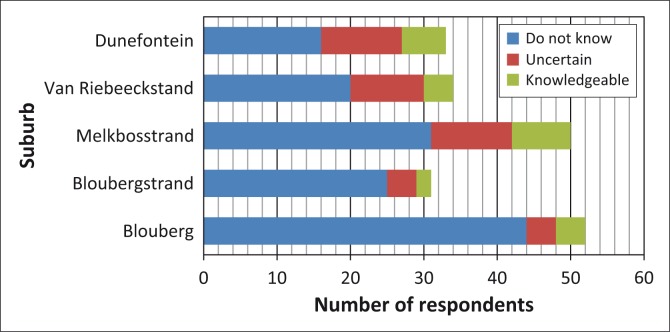
Respondents’ knowledge of location of emergency assembly points and mass-care centres.

Similar trends were observed concerning respondents’ knowledge of and familiarity with notification about general emergencies (Figure 3), the procedure for evacuating and collecting children from school (Figure 4), the roads designated as emergency routes (Figure 5) and the location of emergency assembly points or mass-care centres (Figure 6). Despite the general lack of familiarity with and knowledge of this information, a greater proportion of Duynefontein and Melkbosstrand respondents exhibited some knowledge.

Explanations given by respondents for the poor familiarity amongst the majority of respondents across Ward 23 with information related to general emergencies included the following:

- Some respondents were not aware of the availability or existence of such information.
- Some respondents considered the information too long and boring to read.
- Some acknowledge that ‘I have never got around to reading it’.
- Some believed that the information was unnecessary or would not assist them.
- Respondents did not believe that information was accurate or reliable, or they believed that information was used to create false sense of security.
- Some respondents did not keep the information at hand or lost it.
- Some respondents admit to throwing it away.

During interaction with respondents, it was revealed that they hold serious misconceptions about the potential hazard to which they were exposed. Many respondents appeared to believe that a general-emergency event, or ‘if something went wrong’ at KNPS, would cause an ‘atomic-bomb style’ explosion in which everything around KNPS would be ‘incinerated’ and irradiated for several kilometres within minutes. Therefore, many respondents believed that there was no need to be informed or prepared because they would be unable to escape in time and perish anyway.

### Analysis of respondents’ attitudes and perceptions

#### Perceptions of safety

**FIGURE 7:**
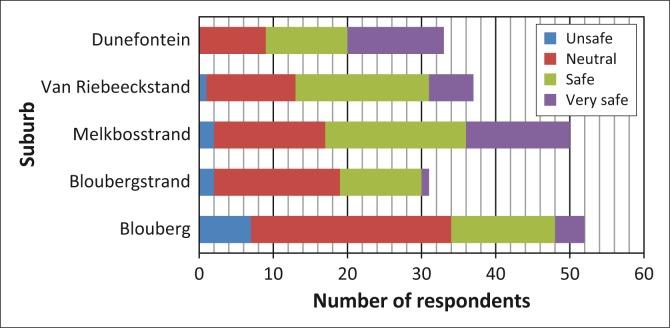
Respondents’ self-rating of their perception of safety residing in the Formal Emergency Planning Zone.

As seen in Figure 7, the majority of respondents across the suburbs stated that they felt neutral or safe (76 and 69, respectively) living in the vicinity of KNPS. Interestingly, most living in suburbs closer to the station held a more positive opinion of their safety than those residing further away.

Their positive opinions were based on their belief that KNPS is well managed and ‘in good hands’ as well as knowing someone (friend, neighbour, spouse, family member or themselves) who either had worked or was currently working at KNPS. Notably, the majority of respondents throughout the ward was more concerned about the threat of crime, car accidents and other everyday hazards to their personal safety. They perceived these hazards to be more likely to occur than a general emergency at KNPS.

#### Perceptions of accessibility, sufficiency and necessity of information

**FIGURE 8:**
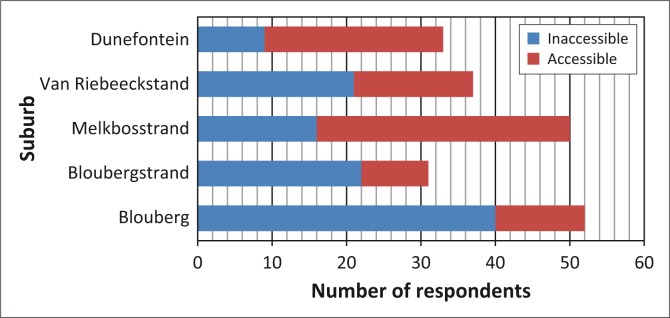
Respondents’ perception of the accessibility and availability of information related to general emergencies.

The majority of respondents situated further away from KNPS, particularly in Blouberg and Bloubergstrand, had a negative perception of the availability and accessibility of information sources related to a general emergency (Figure 8). Many felt that institutions (namely Eskom) had made little or no effort to inform them in any way. Inversely, the majority of respondents residing closer to KNPS felt that information had been made available to the public by stakeholders such as Eskom and CoCT DRMC.

Despite many respondents stating that information was available and accessible, some believed that the information provided was insufficient. Several respondents felt that the information was poor and lacked detail whilst others complained that it was too complicated and confusing. Inversely, others felt that it was sufficiently informative and easy to understand. It was observed that the majority of respondents residing in suburbs closer to KNPS felt that information was sufficiently informative and adequate whilst those further away had mostly an opposite perception.

Whether sufficient information was provided or not, many respondents felt that it was unnecessary to be well informed because of the low probability of such an event, because emergency personnel would instruct them what to do or because there would be no hope of a timeous escape. As a result, many respondents admitted to discarding or throwing away information given to them because the information was deemed to be unnecessary or unimportant.

#### Perceptions on preparation for and evacuation in case of a general emergency

**FIGURE 9:**
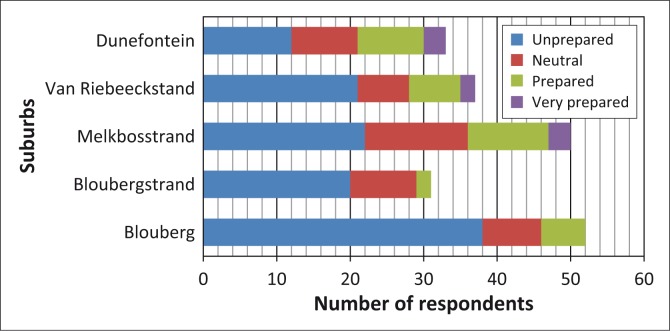
Respondents self-rating their perceived level of preparedness for a general emergency.

In total, 105 of the respondents admitted to being unprepared whilst only 38 considered themselves to be prepared in some way or another (Figure 9).

Their reasons for the lack of (or need for) preparation included:

- They have never thought about it (this perception was generally observed in Bloubergstrand and Blouberg and amongst respondents who were poorly informed).
- KNPS is safe, and the probability of an emergency event is very low, which means that the risk is low.
- The authorities will instruct them on what to do, when to do it and where to go.
- If an event occurs, the situation will be too chaotic and hopeless. This perception was widely held by respondents, concerned about heavy traffic as a result of limited road networks and a large population within the ward as well as the probability of general panic and confusion amongst evacuees.

### Analysing respondents’ practices

#### Disaster preparation and planning

Only 26 respondents had prepared some form of household disaster plan or had a pack of emergency supplies at the ready. Table 3 lists the varied plans and items that these respondents said they would implement and/or take.

**TABLE 3:** List of respondents’ varied disaster plans and supply-pack items.

Disaster plan	Supply pack
Emergency details of family	Water bottles
Checklist of what to take	Canned food
Predetermined place to go to	Packed clothes
Close all windows and doors	Trailer (with several supplies)
Leave immediately	Medical kits (bag for important medication)
Await instructions by authorities	Camping gear
Consult guidelines before deciding what to do	Go-bag (small amounts of each supply in it)

Other general practices and plans concerning preparedness commonly exhibited by these respondents included the following:

- They stored important documentation (identity documents, bank details, birth certificate, et cetera) in one place for easy collecting if need be. Similar provisions were made for personal items such as family memorabilia.
- They kept information sources on general emergencies such as calendars and guidelines in a known and accessible location so as to be consulted in cases of need.
- They ensured that their vehicles have enough petrol to travel more than 25 km away from home.
- Several respondents felt that they were adequately prepared by just reading and familiarising themselves with general-emergency guidelines. Thus, they would not be confused or frightened but would know what to expect and what to do when necessary.

#### Evacuation reaction

**FIGURE 10:**
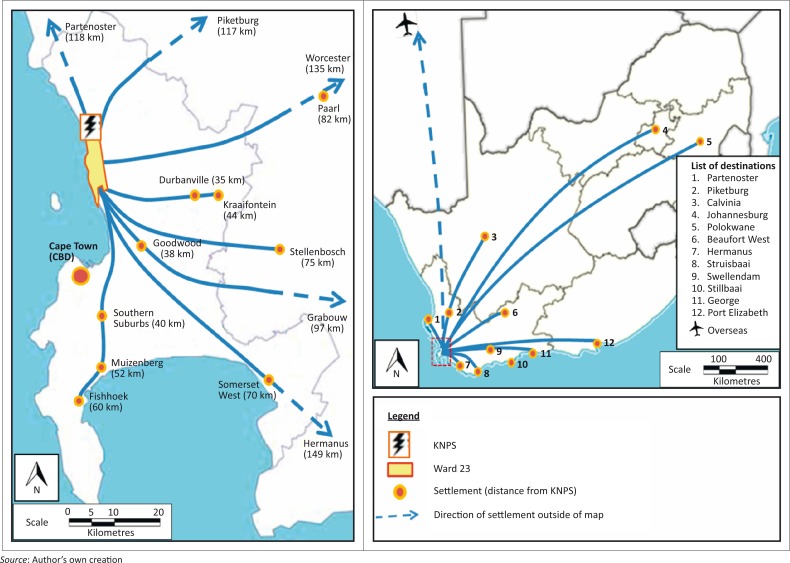
Locations to which respondents would evacuate in the event of a general emergency.

When asked where they would go in the event of a general emergency, many respondents agreed that they would probably travel ‘as far away as possible’, listing locations they perceive to be far away enough (Figure 10). When queried about whether they would wait for instructions before evacuating, these respondents exhibited negative opinions or distrust towards authorities leading an evacuation. Only 16 respondents stated that they would first wait for information about weather conditions and the expected direction of the radiation path and orders from the authorities before deciding on their course of action.

## Discussion

An analysis of the consolidated data reveal that the majority of respondents in Ward 23 have poor levels of knowledge and awareness of information related to general emergencies, and only a few individuals have made any preparations for a potential nuclear hazard and evacuation. Several interconnected factors have been identified as responsible for the level of information awareness, knowledge and preparation. Respondents also exhibited particular perceptions of safety or even fatalism.

### Proximity to hazard affecting levels of knowledge and preparedness

Interestingly, there was no evidence that different demographic characteristics influenced respondents’ levels of awareness, knowledge and preparedness. Whilst this seems strange or unlikely, especially in the setting of a highly heterogeneous country as South Africa, it must be taken into consideration that Ward 23’s population is somewhat homogenous with the majority of respondents interviewed being white, highly educated, employed and living in well-developed formal residential zones. I observed no trends or patterns that suggested a link between particular demographic characteristics such as age, education, gender, class or wealth and respondents’ levels of knowledge and preparedness.

However, it was evident from the data that there were significant differences between these levels of knowledge and preparedness and respondents’ proximity to KNPS. In the literature of geographic vulnerability, it has been commonly observed that those who reside closer to a known hazard tend to demonstrate greater knowledge about the hazard and have a greater probability of developing appropriate preparations for such an event (Lindell & Hwang 2008). A similar trend was observed amongst respondents residing in suburbs closer to KNPS, especially in Duynefontein and Melkbosstrand, who exhibited greater familiarity with and knowledge of emergency-related information. They also had more positive perceptions about the availability, accessibility and sufficiency of said information, and they felt better prepared and safer than respondents residing further away (e.g. Blouberg and Bloubergstrand). The decreased levels of information awareness, knowledge and preparation amongst respondents residing further away from KNPS are probably as a result of a distance-decay effect in which people tend to be less responsive to hazards and exhibit increasingly lower levels of knowledge and preparation with increasing distance from a hazard (Eldridge & Jones 1991).

### Problems with communication and the distribution of information

A multitude of information and material related general emergencies is distributed by Eskom and the CoCT DRMC to the public via several mediums such as by post, online, at local municipal offices, at public libraries or at the KNPS public information centre (City of Cape Town 2012a; Eskom 2013b). Mr Phidza, the Stakeholder management Manager at KNPS, explained in a personal interview that several ‘push and pull’ information strategies have been implemented by Eskom annually. Either updated information is distributed (push strategy) to residents via mediums such as calendars and leaflets, or residents are invited and encouraged to attend (pull strategy) quarterly ‘Public Safety Forum’ meetings, visit the Eskom visitors centre, et cetera. However, despite these efforts to make this information available and accessible to the public, almost half of the respondents were unaware that general-emergency information has been made available and is accessible to the public. Many respondents, especially in Blouberg and Bloubergstrand, claim that there has been little to no effort by stakeholders to provide them with information regarding KNPS or a general emergency. Therefore, relevant stakeholders may need to look into and address this problem concerning information communication and distribution to the respondents.

### Misconceptions regarding a general emergency and exposure to hazards

It was observed that at least a quarter of the respondents believed that a general-emergency event at KNPS could result in an atomic-bomb-style explosion or blast-wave that would cause immediate death and destruction. Thus, many respondents fail to see the need to properly inform or prepare themselves. However, this perception of their exposure to a hazard is greatly mistaken. The materials that nuclear power stations like KNPS use to produce energy are radiological in nature but not explosive (Nosowitz 2011). The belief in an exploding nuclear power station is a common misconception amongst people globally because of the incorrect association of nuclear-energy generation with how nuclear weapons operate (Boisvert 2013). This incorrect assumption amongst the majority of respondents concerning the nature of their nuclear-hazard risk is most likely a consequence of their poor levels of awareness of and familiarity with general-emergency information provided by Eskom and CoCT DRMC.

### Predetermined place to go in case of an emergency

Previous observations at other general emergency situations such as the 1979 Three Mile Island general emergency indicated that the majority of people (even those outside of the designated evacuation zone) would evacuate themselves much further than the authorities stated was actually necessary (Zeigler & Johnson 1984). The cause for this exaggerated evacuation was as a result of evacuees’ fear of radiation and its long-term health effects as well as the misconception or misinformation of hazard exposure and a distrust of authorities (Brumfield 2013; Donn 2013). The majority of respondents in Ward 23 would appear to exhibit behaviour similar to other general-emergency evacuees in order escape the hazard. The respondents’ desire to move so far away, coupled with their poor levels of preparation and knowledge as well as misconceptions concerning a nuclear emergency, appear to be very similar to the extreme evacuation traits observed internationally.

### Sense of security affecting informedness and preparedness

Whilst some respondents see no need to be informed or prepared because they perceive the situation as hopeless, many respondents, especially in Duynefontein and Melkbosstrand, stated that they felt safe with nuclear power and confident in KNPS’s management. Some also indicated that they felt safe because they have a relationship with KNPS staff. This sentiment is not dissimilar to Japan’s population who, prior to the 2011 FNPS general emergency, were confident in the management and operation of its nuclear-power industry, believing the possibility of a nuclear-related disaster to be highly improbable (Funabashi & Kitazawa 2013; Tanaka 2012). Therefore, during the 2011 FNPS general emergency, their false sense of security left them ill prepared and highly vulnerable. The social capital developed from relationships between respondents and KNPS staff may generate greater confidence in and a perception of safety regarding KNPS and its management, but it may ultimately make them less prepared and vulnerable in the event of a general emergency (Claridge 2004).

### Threat of improbable hazards versus everyday hazards

A lack of preparedness by respondents could include the fact that Ward 23 of the CoCT is not exposed to major natural or technological hazards (Van Niekerk & Visser 2010). Hence, with little risk to their livelihoods, respondents have little incentive or need to prepare emergency plans or supplies for such unlikely hazards (Council for Excellence in Government 2006). This coincides with the concept of the ratchet effect according to which respondents may perceive that preparing for an improbable hazard is a waste of resources (Pelling 2003). Respondents generally felt more concerned preparing themselves against more common and probable everyday hazards such as crime and/or car accidents.

### Perception of safety influenced by awareness and knowledge

Whilst it could be supposed that those residing closer to a known hazard would feel less safe than those living further away, the opposite was discovered, namely that those residing closer to KNPS felt significantly safer than those further away. From the analysis of data gathered, it can be assumed that the perceptions of safety are linked to the level of knowledge of respondents concerning information related to nuclear and general emergencies. Several of the previously listed factors such as the high levels of misconception concerning exposure to a nuclear hazard and poor communication and distribution of information, which showed the highest figures in Blouberg and Bloubergstrand, probably contributed to a lack of confidence about safety amongst respondents of these suburbs. Conversely, respondents of Duynefontein and Melkbosstrand felt safer due to increased awareness, communication and familiarity (but not necessarily knowledge of the content) of distributed information.

However, whilst most respondents stated that they generally felt safe residing in the FEPZ on a day-to-day basis, they also expressed major insecurity and an almost fatalistic perception with regard to a general emergency and their evacuation situation. A major concern of respondents is that, in the event of a general emergency, the transport routes leading away from the area will be unable to accommodate the evacuation of the large (and rapidly growing) population residing in the FEPZ before being impacted by the hazard (be it by radiation contamination or an explosion, in some opinions). Similar fears have been expressed in the US and Europe, indicating that a large and growing population in the vicinity of nuclear stations may inhibit evacuation efforts (Donn 2013). Statistics South Africa confirms this, stating that the number of residents and households within Ward 23 has grown by 70% and 75%, respectively, between 2001 and 2011 (Department of Strategic Development Information and GIS 2013). The CoCT DRMC has been working on creating accurate and up-to-date evacuation simulations and emergency exercises for a general-emergency event (Marks *et al*. 2007). Under present conditions, they can evacuate all residents from the FEPZ in the designated 16-hour timeframe. However, they acknowledge that the rapid urban growth within the FEPZ has made it increasingly difficult to ensure that an evacuation can occur within the specified timeframe.

## Conclusion

This study has found the majority of respondents across Ward 23 to be poorly informed and unprepared for a general-emergency event. The consolidation of data revealed evidence of a link between respondents’ increased proximity to KNPS and the increased levels of awareness, knowledge and familiarity concerning general-emergency information, perceptions of the availability, accessibility and sufficiency of said information as well as perceptions of personal preparedness and safety. The two main causes for the general lack of informedness amongst respondents were identified. The first includes that respondents were unaware that information related to general emergencies was available to the public or that they have not received such information. The second cause relates to respondents’ lack of incentive to inform themselves. This disincentive to acquire or familiarise themselves with information can be attributed to (1) a perception of hopelessness and fatalism, (2) misconceptions concerning their exposure to a hazard, (3) the improbability of a threat or their views on their safety and complacency and (4) social capital with KNPS staff and trust in management.

It can be recommended that stakeholders such as Eskom and DRMC improve branding and advertise their information better to make residents of the ward more aware that information exists. Stakeholders should especially focus on correcting misconceptions amongst the public such as the idea of KNPS exploding. Other efforts should include attempting to make information more attractive to incentivise the public to familiarise themselves with the information and to engage with the stakeholders which provide them.
